# News and Events of the Week

**Published:** 1911-06-24

**Authors:** 


### News and Events of the Week.

In the museum of Rheims is a small phial filled with a coloured liquid. Nobody has tasted this liquid, for the waxen plug which corks the bottle is petrified. But it appears that it really is a sample of wine collected from the slopes of the champagne district before the fourth century of our era. It was found in a Gallo-Roman tomb where, as though in a cellar, it has been preserved until the present day.

Communications to be made at the Congress of Pathologists to be held at Turin on October 1 should be sent to the secretary, M. G. Salta, 30 Corso Raffaello, Turin, not later than August 31. The subscription to the Congress is £1. The treasurer is Mr. Vangelti, 15 Via Esposizione, Turin.

The annual general meeting of the Research Defence Society was held on Monday last at the College of Physicians, Lord Cromer, the president, occupying the chair. The annual report, submitted by Mr. Sydney Holland, showed that the Society has made good progress during the past year, and has distributed a tremendous amount of informative literature. Several local branches have been formed. Lord Cromer, in the course of his address, referred to the action recently taken at a meeting of the National Society for the Prevention of Cruelty to Animals (of which he is a vice-president), and said that the Research Defence Society held views in harmony with the older association on the subject of cruelty to animals. The report and balance-sheets were adopted.

Messrs. J. & A. Churchill have in preparation a new annual to be called *Who's Who in Science*. Schedules are being addressed to the scientists whose names may appear, and it is hoped that they will assist the publication by filling in and returning the forms to 7 Great Marlborough Street, London, W., as soon as possible.

The Mission to Lepers in India and the East has been extending its operations. An important new asylum has been built at Fusan, where, for the first time, the numerous outcast lepers of Korea can be received. Altogether £24,000 was expended last year on seventy-seven stations, where 10,250 lepers and children are treated. The Mission now maintains fifty-one asylums, with some 4,000 lepers, in addition to the support and education of nearly 600 untainted children. It is hoped to start work this year in Arabia and Persia, where, we understand, nothing of the kind has yet been done.

It is interesting, if unsatisfactory, to note the fact that the latest announcements of the estates of three medical officers show a grand total of only £12,625. Surgeon-Lieutenant-Colonel Henry Frank Hensman, C.M.G., of Penkridge, Staffs, late 1st Life Guards, Hon. Associate of the Order of St. John of Jerusalem, and from 1900-3 Senior Medical Officer at Shorncliffe, who died on February 21, aged seventy-one, has left estate valued at £1,490 gross. Major George Lamb, M.D., of Edinburgh, late of the Indian Medical Service, Director of the Pasteur Institute, Kassauli, Senior Member of the Plague Commission in India, who died on April 11, aged forty-one, has left personal estate in the United Kingdom valued at £3,945; and Lieutenant-Colonel James Shaw McCutchan, R.A.M.C., of Camden-road, N.W., who died on April 18, aged sixty-five, has left estate of the gross value of £7,190.

